# Philosophical Transactions of the Royal Society of London. Vol. LXXIV. For the Year 1784

**Published:** 1785

**Authors:** 


### SECTION II.

### BOOKS.

I. *Philosophical Transactions of the Royal Society of London. Vol.* LXXIV. *for the year* 1784. 4to. London, 1784. 521 pages.

1. *EXPERIMENTS on Air. By* Henry Cavendish, *Esq.* F.R.S. *and* S. A. — These very ingenious experiments were made principally with a view to find out the cause of the diminution which common air undergoes by phlogistication, and to discover what becomes of the air thus lost or condensed.

It has been supposed that fixed air is either generated or separated from atmospheric air by phlogistication, and that the observed diminution is owing to this cause; Mr. Cavendish's first experiments, therefore, were made with a view to ascertain whether any fixed air is really produced thereby. The results of his inquiries are, that the generation of fixed air is not the general effect of phlogisticating air, and that the diminution of common air is by no means owing to the generation or separation of fixed air from it.

After defcribing fome unfuccefsful attempts to afcertain what becomes of the air loft by phlogiftication, our author proceeds to fome experiments which ferve really to explain the matter.

Mr. Warltire had remarked *, that on firing a mixture of common and inflammable air by electricity in a clofe copper veffel, a lofs of weight was always perceived; and that on repeating the experiment in glafs veffels, the infide of the glafs, though clean and dry before, immediately became dewy. As the latter experiment feemed likely to throw great light on the fubject he had in view, Mr. Cavendifh thought it well worth examining more clofely.

From the experiments he made for this purpofe, it appears that when inflammable and common air are exploded in a proper proportion, almoft all the inflammable air, and near one fifth of the common air, lofe their elafticity, and are condenfed into pure water.

When, inftead of common air, dephlogifticated air was mixed with inflammable air and exploded, the condenfed liquor was found to confift of water united to a fmall quantity of

* See our 2d Vol. p. 102.

nitrous nitrous acid. This experiment was afterwards varied with dephlogiſticated air, procured from different ſubſtances, and mixed with inflammable air in different proportions; and from theſe experiments it appeared, that when a mixture of inflammable and dephlogiſticated air is exploded in ſuch proportion that the burnt air is not much phlogiſticated, the condenſed liquor contains a little acid, which is always of the nitrous kind, whatever ſubſtance the dephlogiſticated air is procured from; but that if the proportion be ſuch that the burnt air is almoſt entirely phlogiſticated, the condenſed liquor is not at all acid, but ſeems pure water; and as, when they are mixed in that proportion, very little air remains after the exploſion, almoſt the whole being condenſed, it follows, that almoſt the whole of the inflammable and dephlogiſticated air is converted into pure water.

Mr. Cavendiſh next offers ſome obſervations, which tend to ſhow that dephlogiſticated air is only water deprived of its phlogiſton; and that inflammable air is either pure phlogiſton, as Dr. Prieſtley and Mr. Kirwan ſuppoſe, or (what he himſelf conſiders as more probable) phlogiſticated water. He thinks, with M. Lavoiſier and Mr. Scheele, that dephlogiſticated ted and phlogifticated air are quite diftinct fubftances, and that common air is a mixture of the two.

There feemed great reafon to think, from Dr. Prieftley's experiments, that both the nitrous and vitriolic acids are convertible into dephlogifticated air; but the experiments in the prefent paper feem to fhow that no part of the acid is converted into dephlogifticated air, and that their ufe in preparing it is owing only to the great power which they poffefs of depriving bodies of their phlogifton. As a ftrong confirmation of this, Mr. Cavendifh obferves, that red precipitate, one of the fubftances yielding dephlogifticated air in the greateft quantity, and which is prepared by means of the nitrous acid, contains in reality no acid.

II. *Remarks on Mr.* Cavendifh's *Experiments on Air. In a Letter from* Richard Kirwan, *Efq. F. R. S. to Sir* Jofeph Banks, *Bart. P. R. S.*

III. *Anfwer to Mr.* Kirwan's *Remarks upon the Experiments on Air. By* Henry Cavendifh, *Efq. F. R. S. and S. A.*

IV. *Reply to Mr.* Cavendifh's *Anfwer. By* Richard Kirwan, *Efq. F. R. S.*—The doctrine maintained by Mr. Cavendifh, that no fixed fixed air is produced in common phlogiſtic proceſſes, being contrary to the opinion delivered by Mr. Kirwan in a former volume* of the Philoſophical Tranſactions, the latter has been induced to vindicate his concluſions on this ſubject. For the particulars of this controverſy we muſt refer our readers to the work itſelf, as it would be impoſſible for us to do juſtice to the arguments of theſe ingenious and reſpectable philoſophers, without exceeding the limits of our Journal.

V. *Thoughts on the conſtituent Parts of Water, and of dephlogiſticated Air; with an account of ſome Experiments on that Subject. By Mr.* James Watt, *Engineer.*—This paper is a very ingenious attempt to prove that water conſiſts of dephlogiſticated air and phlogiſton deprived of part of their latent heat. It is chiefly on this latter point that Mr. Watt differs in his theory from Mr. Cavendiſh, who doubts whether there be any ſuch thing as elementary heat.

VI. *An Account of a new Species of the Bark Tree, found in the Iſland of St. Lucia. By Mr.* George Davidſon.—The ſpecies of Cinchona here deſcribed is nearly about the ſize of a cherry cherry tree, feldom thicker than the thigh, and tolerably ftraight; the wood is light and porous, without any of the bitternefs and aftringency of the bark itfelf.

The leaves are large, oblong, oppofite and plain, preferving (as well as the flowers and feeds) the bitter tafte of the bark.

In the beginning of the rainy feafon (June), the tree puts forth its flowers in fmall tufts; at firft they are white, but afterwards turn purplifh. The ftamina are five in number, with a fingle ftyle. The germen is oblong, bilocular, and furrowed on each fide. The feeds are many, and of the winged kind. The corolla is monopetalous, with its mouth divided into five long fegments.

The bark itfelf is of a lighter red than the red bark, inclining more to the colour of cinnamon. Mr. Davidfon thinks it is more bitter and aftringent than either of the other barks. — He thinks the proper feafon for obtaining it is about the month of March, before the flowers come out.

Infufed in cold water (in which form, or in lime water, he has generally ufed it) it forms a very red tincture, poffeffing the bitternefs and aftringency of the bark very ftrongly. He has likewife likewife given it in fubftance from twenty to thirty grains; but he has never met with any perfon whofe ftomach was able to retain more than twenty grains of it.

Mr. Wilfon, of Henrietta Street, to whom Mr. Davidfon addreffed his defcription of this bark, remarks, (in a letter to Dr. Monro, prefixed to Mr. Davidfon's account) that it is undoubtedly a Cinchona, but not the Cinchona officinalis of Linnæus, from which it differs effentially in its bark in feveral particulars. It has an emetic quality, he obferves, not common to the true bark; breaks more woody and fplintery, and is far more naufeous to the tafte. Its decoction, he tells us, is of a dull Burgundy colour; and its extract, of which he procured four ounces from half a pound of the bark boiled in water, refembles more the bitter of gentian than that of quinquina.

Mr. Wilfon acknowledges himfelf indebted to Sir Jofeph Banks, Bart. for the following botanic character of this new fpecies of bark tree:

" Cinchona floribus paniculatis, glabris; la-
" ciniis linearibus, tubo longioribus; ftami-
" nibus exfertis; foliis ellipticis, glabris."

VII. *A new Method of preparing a Test Liquor to shew the presence of Acids and Alkalis in chemical Mixtures. By Mr.* James Watt, *Engineer.*

VIII. *An extraordinary Case of a Dropsy of the Ovarium, with some Remarks, by Mr.* Philip Meadows Martineau, *Surgeon to the Norfolk and Norwich Hospital.*—Of this and the preceding article we have already given some account *.

Among the other papers in this volume are, *A Meteorological Journal for the year* 1782, *kept at Minehead in Somersetshire, by Mr.* John Atkins.—*An account of some late fiery Meteors, with Observations; in a letter from* Charles Blagden, *M. D. Physician to the Army, Sec. R. S. to Sir* Joseph Banks, *Bart. P. R. S.*—*A Description of the Teeth of the Anarrhichas Lupus* Linnæi, *and of those of the Chætodon nigricans of the same author; to which is added, an attempt to prove that the Teeth of cartilaginous Fishes are perpetually renewed; by Mr.* William André, *Surgeon.*—*Experiments and Observations on the Terra Ponderosa,* &c. *by* William Withering, *M. D.*—*An Attempt to compare and connect the Thermometer for strong Fire, described in Vol.* 72

* See Vol. 5. p. 314.

*of the Philos. Trans. with the common mercurial ones; by Mr.* Josiah Wedgwood, *F. R. S. Potter to her Majesty — An Account of a remarkable Frost on the* 23*d of June,* 1783, *in a Letter from the Rev. Sir* John Cullum, *Bart. F. R. S. and S. A. to Sir* Joseph Banks, *Bart. P. R. S. — Abstract of a Register of the Barometer, &c. at Lyndon in Rutland,* 1783; *by* Thomas Barker, *Esq.* — *An Account of a new Plant of the Order of Fungi, by* Thomas Woodward, *Esq.*

